# Risk and Health Factors for Temporomandibular Disorders Following Radiotherapy for Head and Neck Cancer

**DOI:** 10.1111/joor.70019

**Published:** 2025-07-21

**Authors:** Ellie Saghafi, Kalid Kadhim, Birgitta Johansson‐Cahlin, Charlotte Andrén Andås, Therese Karlsson, Caterina Finizia, Göran Kjeller, Lisa Tuomi

**Affiliations:** ^1^ Department of Oral and Maxillofacial Surgery, Institute of Odontology, Sahlgrenska Academy University of Gothenburg Gothenburg Sweden; ^2^ Clinic of Orofacial Pain Public Dental Service Gothenburg Sweden; ^3^ Department of Orofacial Pain, Institute of Odontology, Sahlgrenska Academy University of Gothenburg Gothenburg Sweden; ^4^ Department of Otorhinolaryngology, Head and Neck Surgery, Institute of Clinical Sciences, Sahlgrenska Academy University of Gothenburg Gothenburg Sweden; ^5^ Department of Otorhinolaryngology, Head and Neck Surgery Sahlgrenska University Hospital Gothenburg Sweden; ^6^ Department of Health and Rehabilitation, Institute of Neuroscience and Physiology, Sahlgrenska Academy University of Gothenburg Gothenburg Sweden

**Keywords:** head and neck cancer, intervention, prevention, radiotherapy, symptom, temporomandibular disorders (TMD), trismus

## Abstract

**Background:**

Temporomandibular disorders (TMD) are multifactorial and complex musculoskeletal conditions frequently associated with pain or dysfunction, including impaired jaw function and pain in the temporomandibular joint, masticatory muscles and/or related structures. Recent studies have found that personality, behaviour and environment can affect the development of TMD.

**Objective:**

This study investigates whether patient‐related factors can predict TMD among patients with head and neck cancer (HNC) after radiotherapy.

**Methods:**

We randomised 58 consecutive patients with squamous cell carcinoma in the head and neck area into two groups: training with a jaw mobiliser once a day or a control group (no training). A comprehensive examination for TMD was conducted at baseline (before oncologic treatment) and 6 and 12 months after radiotherapy. Potential predictors were analysed using linear and logistic regression analyses.

**Results:**

Myalgia was associated with TMD diagnosis at baseline, and jaw exercise lowered the risk of developing myalgia at the follow‐ups. The degree of pain at baseline and jaw exercise were associated with the changes in pain at follow‐ups. Jaw exercise was associated with a lower degree of pain. A high degree of pain at baseline was associated with less pain at follow‐ups. Jaw exercise was associated with a reduced risk of decreased maximal incisal mouth opening post treatment.

**Conclusion:**

No psychosocial or other background factor reduced risk for myalgic pain or pain to the same degree as jaw exercise. Therefore, we recommend using jaw training to prevent the deterioration of maximal incisal opening and the development of pain associated with TMD.

## Graphical abstract

To investigate whether patient‐related factors can predict temporomandibular disorders (TMD) among patients with head and neck cancer (HNC) after radiotherapy. 58 patients with squamous cell carcinoma of the head and neck Randomised into two groups: jaw mobilizer training versus control. TMD assessments at baseline, 6 months and 12 months post‐radiotherapy. Linear and logistic regression analyses of predictors. Jaw exercise was associated with reduced myalgia, degree of pain, and limited mouth opening. No psychosocial or background factor showed similar protective effects. Jaw training is recommended to prevent TMD‐related complications post‐radiotherapy.

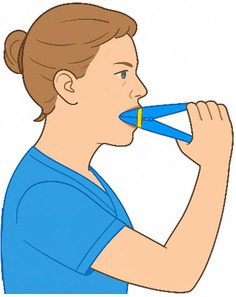

## 1 Introduction

TMD, multifactorial and complex musculoskeletal conditions of the face, jaw and temporal regions, is associated with pain or dysfunction such as impaired jaw function, and pain in the temporomandibular joint (TMJ), masticatory muscles and/or related structures as well as associated headaches. Recent studies have found that psychosocial factors such as personality, behaviour, and environment influence the development of TMD [1, 2, 3]. Other studies have shown that oral parafunctions may play a role in the aetiology of TMD [4, 5, 6]. The prevalence of TMD symptoms in the general population is as high as 5%–15% [7]; however, Ryan et al. (2019), in a systematic review of TMD epidemiology in the general population, highlighted significant variability in reported prevalence rates, ranging between 1% and 75%, a disparity attributed to differences in study methodologies, diagnostic criteria, and population characteristics. The authors emphasise the need for standardised diagnostic criteria in future research to obtain more consistent prevalence estimates and to better understand the aetiology of TMD. The systematic review produced several key findings: the prevalence of TMD peaks between the ages of 25 and 45; women are more affected than men; and an upward trend in the prevalence of TMD within the general population has been observed in recent decades [8].

Head and neck tumours, a heterogeneous group of malignancies, can severely affect many important basic human functions that may affect quality of life [9, 10]. Tumours in this region as well as the consequences of their treatment can impair essential functions such as speech, swallowing, breathing, smell, and taste, which can result in pain and oral dysfunction [10, 11].

Radiation therapy side effects to the head and neck include xerostomia, mucositis, dysphagia, osteoradionecrosis, and trismus (i.e., reduced mouth opening ability) [11, 12].

The prevalence of TMD symptoms is as high as 39% in head and neck cancer (HNC) patients pretreatment [13, 14]; this prevalence significantly increases after cancer treatment, between 59% and 94% at 6 months and 81% at 12 months post treatment [14].

Although there is a need to evaluate prevention efforts regarding symptoms indicative of TMD in HNC patients, little is known about risk factors such as psychosocial influences on the development of TMD in this patient population. A recent meta‐analysis of randomised controlled trials found that jaw exercises could improve mouth opening over the short (5–10 weeks) and long‐term (3 months) post radiation [15]. In a recent study by this research group, preventive jaw exercise not only reduced the deterioration of mouth‐opening ability but also reduced the prevalence of myalgia compared to a control group [16]. Those who report pain in the masticatory system before treatment are at a higher risk of developing radiation‐induced trismus 6 months post radiation [13]. It is possible that patients with TMD before cancer treatment are at higher risk of being affected by treatment‐related side effects [17, 18]. It is still unknown whether there are other possible factors that may predict changes and the incidence of TMD symptoms in patients with HNC undergoing oncological treatment.

## 2 Objective

This prospective study identifies possible risk or health factors that may predict changes in the incidence of TMD symptoms such as myalgia, the intensity of muscular pain and reduced mouth‐opening ability in patients with HNC 6 and 12 months after oncological treatment. We hypothesise that pretreatment patient‐related factors may predict a higher risk for TMD development among patients with HNC.

## 3 Material and Methods

### 3.1 Subjects

The participants were selected from attendees of the weekly multidisciplinary tumour board meetings held at Sahlgrenska University Hospital (Gothenburg, Sweden) between 2020 and 2022. Eligible individuals included adult patients newly diagnosed with tumours of the oropharynx, hypopharynx, and larynx who underwent curative external beam radiation therapy (EBRT) with or without concomitant chemotherapy. Exclusion criteria comprised prior surgery for HNC (excluding tonsillectomy or diagnostic sample excision), previous radiation therapy or other HNC treatments, tracheostomy, neurological or neuromuscular disorders, cognitive impairments preventing questionnaire completion, history of or current trismus which is maximal incisal opening (MIO) ≤ 35 mm, inadequate dental status (preventing jaw trainer use) or general health conditions that exclude exercise participation. Eligible patients were contacted and invited to join the study. Those who consented were randomly assigned to either the preventive jaw training group or the control group according to optimal allocation via Pocock's sequential randomisation method. Patient data from the study were entered into a secure registry and processed electronically using SPSS version 28.0.1.0 (142) with access restricted to the principal investigator and co‐investigators only. All data are confidential and protected from unauthorised access.

All data will be retained for 25 years to ensure the long‐term preservation of research results and to enable future verification or reanalysis. De‐identification is completed through separation of the code key. Digital files will be deleted at the conclusion of the study, and remaining documents will be securely archived at the academic institution, both during the study and after its completion.

### 3.2 Experimental Design

This single‐blinded randomised controlled trial included a baseline evaluation of jaw system pain and function using standardised Diagnostic Criteria for Temporomandibular Disorders (DC/TMD) followed by assessments 6 and 12 months after treatment. Computerised randomisation employed optimal allocation via Pocock's sequential randomisation method, considering tumour characteristics, age, sex, comorbidities (evaluated with the Adult Comorbidity Evaluation‐27), and MIO. Anticipating potential dropouts, 58 patients were targeted for inclusion, and power calculations were based on prior research findings.

The intervention involved both active and passive jaw exercises utilising a mechanical device known as the JawTrainer. Passive training entailed stretching the jaw with the device for 30 s, repeated three times with 20–25 s of rest between each stretch. Active exercises required the patient to bite against the resistance of the JawTrainer for a few seconds, performed in five repetitions. This regimen was conducted once daily, beginning at diagnosis, continuing throughout radiotherapy (RT), and up to 1 month following the completion of treatment. The participants were recommended to continue the training programme for at least three times/week during the whole follow‐up period.

The primary analyses of the effect of preventive jaw exercise on TMD are described in our previous article: 39% of participants had a TMD diagnosis at baseline and 64% and 48% of participants had a TMD diagnosis at 6 and 12 months, respectively [16]. The control group experienced greater declines in MIO compared to the intervention group, with significant differences at both the 6‐ and 12‐month follow‐ups. The intervention group had a high compliance rate (92% at 6 months and 80% at 12 months) with the prescribed jaw exercises and reported no adverse effects [16].

At baseline, the subjects answered questionnaires that are part of the comprehensive Axis II instruments in the DC/TMD. DC/TMD is used when indicated by clinical specialists or researchers to obtain a more comprehensive evaluation of psychosocial functioning [19]. The instruments were chosen based on prior knowledge of possible risk factors that influence the incidence and continuation of TMD symptoms in HNC patients [13, 17, 18]. In this study, the results from the instruments were then related and compared to the outcome of the TMD diagnosis from the DC/TMD examination.

### 3.3 Cancer Treatment

All patients underwent treatment following the national cancer treatment programme [20]. Curative EBRT was administered using volumetric modulated radiation therapy (VMAT) with a moderately accelerated fractionation schedule, typically totalling 68 Gy in 2 Gy fractions once or twice daily for six treatments per week. Most patients also received concomitant chemotherapy, primarily consisting of six cycles of Cisplatin, although eight patients did not undergo chemotherapy [21].

### 3.4 Examination

At baseline, the 6‐ and 12‐month follow‐up, the participants received a comprehensive examination based on DC/TMD [19], carried out by one of two examiners (authors ES or KK). The study design employed a single‐blind approach, as the examiners were unaware of the participants' group allocation (intervention or control). To ensure consistency, the two examiners underwent calibration for both intra‐ and inter‐rater reliability in accordance with the DC/TMD Training and Calibration Protocols recommended by the International RDC/TMD Consortium [19]. The examination protocol encompassed 10 items, including pain location, incisal relationship, jaw movement measurements, temporomandibular joint noise and muscle palpation. (MIO) was measured in mm using a ruler, and pain was assessed using muscle and TMJ palpation. A short, focused Symptom Questionnaire (DC/TMD SQ) captured pain characteristics, jaw function history, and headache. DC/TMD SQ enhances diagnostic accuracy and facilitates consistent data collection in both clinical and research settings [19]. Six common TMD diagnoses were evaluated, and comprehensive Axis II instruments were used to assess psychosocial functioning as they are all validated self‐administered tools that include measures for a more comprehensive assessment of emotional functioning to evaluate emotional distress, pain‐related disability, and psychosocial functioning [19]. The Patient Health Questionnaire‐9 (PHQ‐9) was used to assess depression severity and functions as a screening, diagnostic, and symptom‐tracking measure [22]. PHQ‐9 evaluates the nine depression criteria, scoring each item from 0 (not at all) to 3 (nearly every day), with a total score range of 0–27. Depression severity is categorised as mild [5], moderate [10], moderately severe [15] and severe [20]. Axis II also includes the Generalised Anxiety Disorder‐7 (GAD‐7), which assesses the severity of generalised anxiety disorder [23]. This seven‐item questionnaire evaluates symptom severity over the past 2 weeks using response options ranging from 0 (not at all) to 3 (nearly every day). The total score ranges from 0 to 21, with cutoff scores of 10 and 15 indicating moderate and severe anxiety, respectively. The Patient Health Questionnaire‐15 (PHQ‐15), designed to assess the severity of somatic symptoms, includes 15 items covering common physical symptoms experienced by patients [24]. Each symptom is rated on a scale from 0 (not bothered at all) to 2 (bothered a lot), yielding a total score between 0 and 30. Somatic symptom severity is categorised as minimal (0–4), low [5, 6, 7, 8, 9], medium [10, 11, 12, 13, 14] and high [15, 16, 17, 18, 19, 20, 21, 22, 23, 24, 25, 26, 27, 28, 29, 30]. The PHQ‐15 is used in both medical and mental health settings to assess somatization and to guide treatment planning. The Perceived Stress Scale (PSS‐10) comprises 10 items used to evaluate stress levels in individuals aged > 12 years. Widely used, PSS‐10 measures the perception of life situations as stressful: < 9 = low stress; 9–16 = under moderate stress; 16–23 = over moderate stress; 23–30 = high stress; and 30–40 = very high stress [25, 26].

The Graded Chronic Pain Scale, a short, reliable and validated instrument that assesses pain intensity and pain‐related disability, has two subscales: Characteristic Pain Intensity (CPI) and Pain‐disability Rating. The CPI reliably measures pain intensity and is comprised of the mean value of three measures—the intensity of current, average, and worst clinical pain over the past month with scores ranging from 0 to 10 using a Numeric Rating Scale (NRS) where 0 is no pain and 10 is the worst possible pain, with ≥ 5 considered high intensity pain. The Pain‐disability Rating is based on the number of days that pain interferes with activity and on the extent of interference with social, work, or usual daily activities. High pain and high interference or moderate to severe disability (classified as Grades 3 or 4) are interpreted as a disability due to pain that warrants further investigation and suggests that pain is significantly affecting an individual [27, 28].

At baseline, participants were also asked to rate their perceived general health from 1 (very bad) to 7 (excellent health) by answering the following question: ‘How would you describe your overall health during the past week?’ and ‘Select the level that suits you best’.

### 3.5 Statistics

For categorical variables, data were presented as *n* (%). For continuous variables, mean (SD), median (min; max), and sample size (*n*) were reported. Group comparisons were conducted using Fisher's Exact test for dichotomous variables, the Chi‐squared test for non‐ordered categorical variables, and the Mann–Whitney *U*‐test for continuous or ordinal variables. Spearman's correlation was used to test correlation between two continuous or ordinal variables. Kruskal–Wallis test was used to test the difference between more than two independent groups (tumour location). Two‐sided *p* value was used for all analyses and *p* < 0.05 was considered statistically significant. The analysis included baseline patient and treatment characteristics such as comorbidities, age, gender, tumour location, preventive jaw exercise, and tumour stage (I–IV) and the baseline patient‐reported instruments. In addition, the analysis used the presence of the six most common TMD diagnoses according to DC/TMD (myalgia, arthralgia, disc displacement with reduction, and disc displacement reduction, headache attributed to TMD, and degenerative joint disease). The primary endpoint in the analysis was the occurrence of the most common TMD diagnosis, myalgia, and changes in the degree of pain using the NRS from baseline to the 6‐ and 12‐month follow‐ups. In addition, the analysis included the endpoints the incidence of trismus and degree of mouth opening from baseline to the 6‐ and 12‐month follow‐ups.

Multivariable analysis was performed with linear and logistic regression. The former was used when the dependent variable showed differences in NRS and MIO between baseline and 6 months and 1 year, and the latter was used when the dependent variable was myalgia and trismus at 6 months and 1 year. Independent variables were baseline variables such as age, gender, tumour location, and preventive jaw exercise, that had a *p* value of *p* < 0.05 in the univariable analysis with non‐parametric tests described above. The regression analyses were performed with the continuous variables (dependent and independent) as they were and with them transformed to normal distribution, and the result was similar.

### 3.6 Ethics

This study was approved by the Swedish Ethical Review Authority (1151‐18 and 2019‐00752). The study was conducted according to the Declaration of Helsinki and all participants gave their written informed consent to participate.

## 4 Results

**FIGURE 1:**
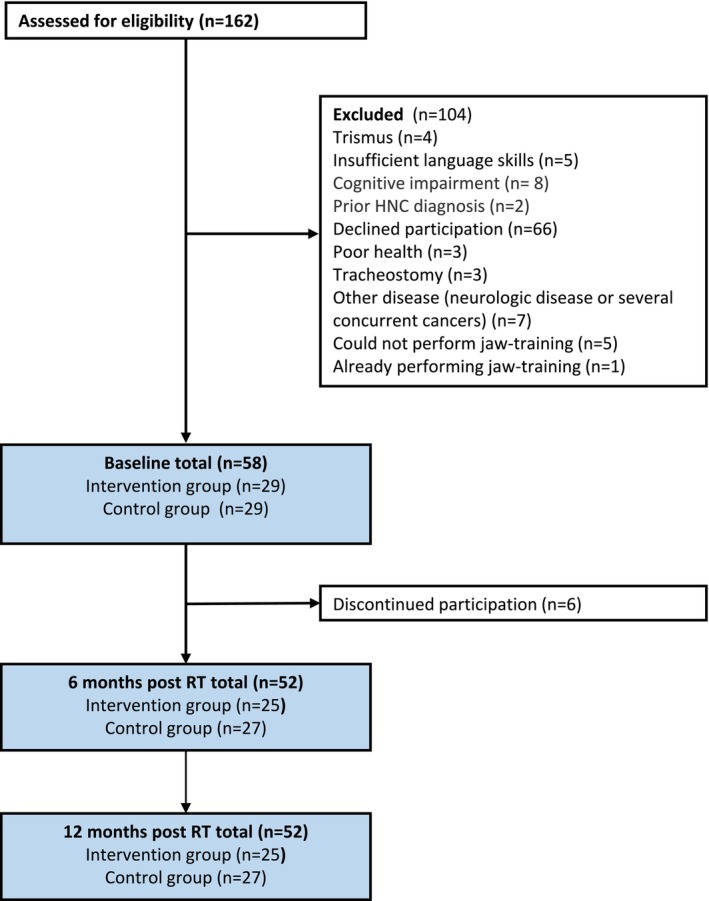
Flow chart showing assessment for eligibility.

From January 2020 to November 2022, a total of 162 patients were assessed for eligibility. Of these, 104 were deemed ineligible and excluded from the study. The remaining 58 patients consented to participate and were randomly assigned to either a preventive exercise group (*n* = 29) or a control group (*n* = 29). All randomised participants completed the baseline examinations. At the 12‐month follow‐up, 52 participants remained enrolled in the study. See Figure 1 for flowchart.

Table 1 presents the baseline characteristics of the patients. The mean age was 67 years. The most common location of the tumour for the male participants was the tonsils. No statistically significant differences were observed between the two treatment groups except for PHQ‐9: the control group had lower values (i.e., lower degree of depression). Dropout analyses were conducted by comparing the characteristics of patients who did not complete the study with those who remained enrolled in the study with no significant differences in any of the variables reported.

**TABLE 1:** Baseline variables and descriptive characteristics at baseline for all participants in the study and comparison between intervention and control group.

Baseline variable	Total *n* = 58	Intervention *n* = 29	Control *n* = 29	*p* value comparison intervention control
	Mean (SD)	Mean (SD)	Mean (SD)	
	Median (min; max)	Median (min; max)	Median (min; max)	
Age (years)	67.2 (9.8) 67 (42; 85)	68.5 (9.0) 68 (46; 83)	65.8 (10.6) 67 (42; 85)	> 0.30
	(%)^a^	(%)^a^	(%)^a^	
Sex				
Male	39 (67.2)	20 (69.0)	19 (65.5)	> 0.30
Female	19 (32.8)	9 (31.0)	10 (34.5)	
Comorbidity according to ACE‐27				
None—Mild	45 (77.6)	22 (75.9)	23 (79.3)	> 0.30
Moderate—Severe	13 (22.4)	7 (24.1)	6 (20.7)	
Tumour location				
Tonsil	34 (58.6)	14 (48.3)	20 (69.0)	0.13
Base of tongue	19 (32.8)	13 (44.8)	6 (20.7)	
Oropharynx	5 (8.6)	2 (6.8)	3 (10.3)	
Tumour stage				
I	30 (51.7)	15 (51.7)	15 (51.7)	> 0.30
II	4 (6.9)	3 (10.3)	1 (3.4)	
III	14 (24.1)	8 (27.6)	6 (20.7)	
IV	10 (17.2)	3 (10.3)	7 (24.1)	
Radiotherapy	58 (100.0)	29 (100.0)	29 (100.0)	
Chemotherapy (concomitant)	50 (86.2)	26 (89.7)	24 (82.8)	> 0.30
DC/TMD diagnoses		*n* = 29	*n* = 29	
No diagnosis	35 (60.3)	17 (58.6)	18 (62.1)	> 0.30
Myalgia	6 (10.3)	3 (10.3)	3 (10.3)	
Arthralgia	3 (5.2)	2 (6.9)	1 (3.4)	
Disc displacement with reduction	11 (19.0)	6 (20.7)	5 (17.2)	
Disc displacement without reduction	2 (3.4)	1 (3.4)	1 (3.4)	
Headache attributed to TMD	0 (0.0)	0 (0.0)	0 (0.0)	
Degenerative joint disease	7 (12.1)	4 (13.8)	3 (10.3)	
PHQ‐9	5.0 (55.9) 3.0 (0; 24)	6.3 (6.2) 5.0 (0; 24)	3.7 (5.3) 1.5 (0;19)	**0.029**
GAD‐7	3.5 (4.3) 2.0 (0; 16)	3.9 (4.1) 2.0 (0; 16)	3.0 (4.6) 1.0 (0;16)	0.14
PSS‐10	11.3 (8.9) 9.0 (0; 37)	11.7 (9.3) 8.5 (0; 37)	10.8 (8.7) 10.0 (0; 34)	> 0.30
PHQ‐15	4.9 (3.9) 4.0 (0; 17)	5.5 (4.3) 4.0 (0; 17)	4.2 (3.4) 3.5 (0; 13)	0.26
Global health	4.9 (1.6) 5.0 (1; 7)	4.8 (1.6) 5.0 (1; 7)	5.0 (1.4) 5.0 (2; 7)	> 0.30
NRS	1.3 (1.8) 0 (0; 7)	1.3 (2.1) 0 (0; 7)	1.4 (1.5) 1 (0; 5)	> 0.30
MIO	48.1 (6.2) 48 (36; 61)	47.7 (6.4) 47 (36; 60)	48.4 (6.1) 50 (40; 61)	> 0.30

*Note:* A higher mean value on PHQ‐9, GAD‐7, PSS‐10, and PHQ‐15, means a higher degree of the symptoms/disorders. A higher mean value on NRS means a higher degree of pain. A higher mean value on Global Health means better health status. A participant can have more than one TMD diagnosis. Bold values means statistically significant.

Abbreviations: ACE‐27, Adult Comorbidity Index; DC/TMD, Diagnostic Criteria for Temporomandibular Disorders; GAD‐7, Generalised Anxiety Disorder; MIO, Maximal Incisal Opening; NRS, Numeric Rating Scale for Measuring Pain Intensity; PHQ‐1, Patient Health Questionnaire for Physical Symptoms; PHQ‐9, Patient Health Questionnaire for Depression; PSS‐1, Perceived Stress Scale; TMD, Temporomandibular Disorders.

^a^ Percentages rounded (i.e., does not always sum to 100).

At baseline, the mean value for the PHQ‐9 question regarding mild depression was 5 (Table 1), but the intervention group had a significantly greater mean value for a higher degree of depression (*p* = 0.029) (Table 1). In the GAD‐7 questionnaire, the mean value for anxiety was 3.5 (i.e., a low level of anxiety). In the PSS‐10 questionnaire, the mean value for stress level was 11.3 (i.e., stress levels under moderate). In the PHQ‐15 questionnaire, the mean value for somatic symptoms was 4.9 (i.e., low somatic symptom). The mean value for the answers to the question regarding general health was 4.9 (i.e., good to very good perceived general health).

### 4.1 Risk Factor Models

Univariable associations between baseline variables and outcomes were investigated. The variables found to be statistically significant associated were then used in a multivariable regression analysis to identify potential independent risk factors that could predict the prevalence of myalgia, changes in pain intensity measured with NRS, and the degree of mouth‐opening and trismus in patients with HNC at 6 and 12 months post radiotherapy (RT). See Table 2 for the univariable analysis and Table 3 for the multivariable regression analysis.

**TABLE 2:** Univariable *p* values for associations between baseline variables and changes from baseline in NRS and changes in MIO, myalgia status and trismus status at 6 months and 12 months.

Baseline variables	NRS baseline‐6M	NRS baseline‐12 M	MIO baseline‐6M	MIO baseline‐1Y	Myalgia 6M	Myalgia 12M	Trismus 6M	Trismus 12M
Age	0.39	0.23	0.59	0.97	0.46	0.12	1.00	0.36
Gender (0/1)	0.094	0.24	0.11	0.34	0.76	0.19	0.45	1.00
Tumour location	**0.012**	0.075	0.24	0.37	0.30	1.00	0.19	0.91
Tumour stage (I–IV)	0.98	0.22	0.32	0.86	0.30	1.00	0.32	0.85
NRS	**0.008**	**< 0.001**	0.28	0.61	**0.028**	0.45	0.27	0.44
PHQ‐9	0.48	0.13	0.23	0.16	**0.046**	0.082	0.49	0.93
GAD‐7*	0.98	0.27	0.36	0.14	0.22	0.29	0.37	0.43
PSS‐10	0.99	0.35	0.48	0.25	0.15	0.24	0.36	0.53
PHQ‐15	0.71	0.077	0.43	0.16	0.094	0.36	0.47	0.82
(MIO)* in mm	0.86	0.61	0.34	0.26	0.18	0.15	0.20	0.26
DC/TMD diagnosis	0.62	0.31	0.37	0.26	**0.040**	0.33	0.47	0.70
ACE‐27	0.71	0.97	0.54	0.63	1.00	1.00	1.00	1.00
Global health	0.37	0.34	0.38	0.20	0.52	0.088	0.36	0.84
Jaw exercise	**0.012**	0.22	**< 0.001**	**< 0.001**	**< 0.001**	**0.004**	0.073	0.42
Myalgia BL	0.80	0.16	0.98	0.55	**0.010**	0.60	1.00	0.53

*Note:* Group comparisons were conducted using Fisher's Exact test for dichotomous variables, the Chi‐squared test for non‐ordered categorical variables, and the Mann–Whitney U‐test for continuous variables. Spearman's correlation was used to test correlation between two continuous or ordinal variables. Kruksal–Wallis test was used to test difference between more than two independent groups (tumour location). Two‐sided *p* values were used for all analyses and *p* < 0.05 was considered to be statistically significant. Thes following variables were excluded from the analysis due to low statistical power: Arthralgia, Disc displacement without reduction, and Headache attributed to TMD. The limited number of observations for these diagnoses affected the ability to draw statistically reliable conclusions. Instead, the category DC/TMD diagnosis was included, which also captured these cases. Bold values means statistically significant.

Abbreviations: ACE‐27, Adult Comorbidity Index; BL, Baseline; DC/TMD, Diagnostic Criteria for Temporomandibular Disorders; GAD‐7, Generalised Anxiety Disorder; M, months; MIO, Maximal Incisal Opening; NRS, Numeric Rating Scale for measuring pain intensity; PHQ‐1, Patient Health Questionnaire for Physical Symptoms; PHQ‐9, Patient Health Questionnaire for Depression; PSS‐1, Perceived Stress Scale; TMD, Temporomandibular Disorders.

**TABLE 3:** Multivariable *p* values for associations between baseline variables and changes from baseline in NRS and changes in MIO, myalgia and trismus status at 6 months and 12 months.

Baseline variables	NRS baseline‐ 6M		NRS baseline‐12M		MIO baseline‐6M		MIO baseline‐12M		Myalgia 6M		Myalgia 12	
	β (95% CI)	*p*	β (95% CI)	*p*	β (95% CI)	*p*	β (95% CI)	*p*	OR (95% CI)	*p*	OR (95% CI)	*p*
Tumour location												
Tonsil	1.00											
Base of tongue	−1.06 (−2.54, 0.42)	0.17										
Oropharynx	1.95 (−0.90, 4.80)	0.19										
NRS	**−0.83 (−1.22, −0.44)**	**< 0.001**	**−1.02 (−1.33, −0.71)**	**< 0.001**					1.31 (0.84, 2.03)	0.23		
PHQ‐9									1.01 (0.88, 1.15)	0.89		
Jaw‐exercise	**−2.13 (−3.47, −0.79)**	**0.003**			**5.46 (1.88, 9.04)**	**0.004**	**4.84 (1.62, 8.06)**	**0.005**	**24.39 (4.37, 142.86)**	**< 0.001**	**9.17 (1.80, 47.62)**	**0.008**
Any DC/TMD diagnosis									**6.62 (1.22, 35.71)**	**0.028**		
Myalgia									—	—		

*Note:* Multivariable linear regression when β is presented. Multivariable logistic regression when OR is presented. Bold values mean statistically significant.

Abbreviations: DC/TMD, Diagnostic Criteria for Temporomandibular Disorders; NRS, Numeric Rating Scale for Measuring Pain Intensity; PHQ‐9, Patient Health Questionnaire for Depression.

#### 4.1.1 Changes in Pain Measured With NRS at 6‐Month Follow‐Up

Univariable analysis showed that the tumour location (*p* = 0.012), the NRS at baseline (*p* = 0.008), and intervention with jaw exercises (*p* = 0.012) were statistically significantly associated with change in pain intensity measured with NRS at the 6‐month follow‐up. In the multivariable linear regression analysis, only the NRS at baseline (−0.8 [95% CI −1.2, −0.4, *p* < 0.001]) and jaw exercises (−2.1 [95% CI −3.5, −0.8, *p* = 0.003]) were significantly associated with change in pain intensity. Those who performed jaw exercises during oncologic treatment decreased 2.1 units more in NRS between baseline and the 6‐month follow‐up than the control group. That is, those who performed jaw exercises had a greater improvement in the degree of pain than the control group. One unit higher NRS at baseline resulted in a decrease of 0.8 units more in NRS at the 6‐month follow‐up, a finding that indicates that those with a higher degree of pain at baseline had greater improvement in pain at the 6‐month follow‐up.

#### 4.1.2 Changes in Pain Measured With NRS at 12‐Month Follow‐Up

Univariable analysis showed that NRS at baseline (*p* < 0.001) was the only variable statistically significantly related to a change in NRS at the 12‐month follow‐up (−1.0 [95% CI −1.3, −0.7, *p* < 0.001], linear regression model). One unit higher on the NRS at baseline resulted in a decrease of 1.0 unit more in NRS at the 12‐month follow‐up. That is, those with a higher degree of pain at baseline had a greater improvement in pain at the 12‐month follow‐up.

#### 4.1.3 Myalgia at 6‐Month Follow‐Up

Univariable analysis showed that jaw exercise (*p* < 0.001), NRS at baseline (*p* = 0.028), PHQ‐9 (*p* = 0.046), and TMD diagnosis at baseline (*p* = 0.040) were statistically significantly related to the risk of a myalgia diagnosis at the 6‐month follow‐up. In the multivariable logistic regression analysis, jaw exercise (OR 24.4, 95% CI [4.4, 142.9], *p* < 0.001) and TMD diagnosis at baseline (OR 6.62, 95% CI [1.2, 35.7], *p* = 0.028) were the only variables related to the risk of myalgia at the 6‐month follow‐up. That is, those who did not perform jaw exercises had a higher risk of having myalgia at the 6‐month follow‐up than the intervention group. Patients with any TMD diagnosis at baseline had a higher risk of having myalgia at the 6‐month follow‐up than those without TMD diagnosis. Myalgia at baseline was highly predictive of myalgia at 6 months (*p* = 0.010). However, logistic regression was not possible with myalgia at baseline as an independent variable and myalgia at 6 months as a dependent variable. This limitation might be explained by myalgia at baseline and at 6 months forming a complete or perfect separation statistically [29].

#### 4.1.4 Myalgia 12‐Month Follow‐Up

Univariable analysis showed that jaw exercise (*p* < 0.004) was the only variable found to be statistically significantly related to an increased risk of a myalgia at the 12‐month follow‐up (OR 9.2) (95% CI [1.8, 47.6], *p* = 0.008). That is, those who did not perform jaw exercises had a higher risk of developing myalgia at the 12‐month follow‐up than the intervention group.

#### 4.1.5 Changes in MIO and Trismus 6 Months Follow‐Up

Univariable analysis showed that jaw exercise (*p* < 0.001) was the only variable related to a change in MIO at the 6‐month follow‐up (OR 5.5) (95% CI [1.9, 9.0], *p* = 0.004). The control group decreased 5.5 mm more in MIO between baseline and the 6‐month follow‐up on average than the jaw exercise group. No variable had a statistically significant association with the development of trismus (MIO ≤ 35 mm) between baseline and the 6‐month follow‐up.

#### 4.1.6 Changes in MIO and Trismus at the 12‐Month Follow‐Up

Univariable analysis showed that jaw‐exercise (*p* < 0.001) was the only variable statistically significantly related to a change in MIO at the 12‐month follow‐up (OR 4.8) (95% CI [1.6, 8.1], *p* = 0.005). The control group decreased 4.8 mm more in MIO between baseline and the 12‐month follow‐up on average than the jaw‐exercise group. No variable was statistically significantly associated with change in trismus between baseline and the 12‐month follow‐up.

## 5 Discussion

The primary objective of this study was to prospectively identify potential risk factors that could predict changes in and the occurrence of TMD symptoms, such as myalgia, the intensity of pain, and limited mouth opening, in patients with HNC 6 and 12 months following oncological treatment. Jaw exercise given during RT seems to be the main factor related to the reduction of the risk of developing myalgia and the intensity of pain.

The regression analysis indicated that jaw exercise was the strongest predictor of reduction of pain intensity measured with NRS, suggesting that other predictors may have a diminished or negligible effect. This finding is in line with studies that show jaw exercise can reduce pain intensity both in HNC patients and in the general population [16, 30]. This outcome could be explained by several key factors, for instance factors related to the mechanisms of musculoskeletal adaptation and pain modulation. Jaw exercise promotes neuromuscular re‐education, improving coordination and reducing dysfunctional muscle activity and therefore decreasing myalgia and pain intensity [31]. Repeated movement and controlled loading of the temporomandibular joint (TMJ) and associated muscles may reduce pain sensitivity through mechanotransduction, where mechanical stimulation leads to physiological adaptations that decrease pain perception [32, 33]. Muscle activity enhances circulation (i.e., oxygen delivery to affected tissues), which can reduce inflammation and facilitate the removal of metabolic byproducts associated with pain [34]. Engaging in structured exercises may also have a psychological component, promoting a sense of control over symptoms, reducing fear‐avoidance behaviours and enhancing patient adherence to rehabilitation strategies [35, 36].

The multivariable analysis showed that the other factor related to the reduction of pain intensity was the intensity of pain before treatment. Here, the patients who had a higher level of pain intensity at baseline showed a larger decrease in pain intensity at the 6‐ and 12‐month follow‐ups. A higher value on the NRS allows for a greater possibility of decrease since an NRS score of 1 at baseline only allows for a one unit decrease compared to an NRS 2, which allows for a two unit decrease, an NRS 3, which allows for a three unit decrease, etc. A previous study found that understanding the meaning of changes in the NRS and pain reduction is indispensable for the proper interpretation of the effectiveness of pain treatment and that in patients with acute pain both the meaning of changes in NRS and the meaning of percent pain reduction depend on baseline pain intensity [37]. It is possible that the patients in this cohort with a higher NRS at baseline were patients with pain caused by the actual tumour rather than myofascial pain related to general TMD. Therefore, it is possible that the decrease in pain intensity was greater post treatment due to factors related to the recession of the malignant disease.

For TMD diagnosis myalgia, which is a muscular pain diagnosis, the multivariate analysis revealed that those who did not perform jaw exercise had a higher risk of developing myalgia at the 6‐month follow‐up than the intervention group. The other factor was any TMD diagnosis at baseline, which was associated with a higher risk of having myalgia at the 6‐month follow‐up. This finding agrees with studies that found that pain before treatment is a predictor for maintaining and developing myalgia [17, 18]. At the 12‐month follow‐up, only jaw exercise was associated with a reduced risk of developing myalgia.

The regression analysis showed that only jaw exercise was associated with a reduced risk of MIO decrease both at the 6‐ and 12‐month follow‐ups. This finding is in line with a recently published meta‐analysis, which found that jaw exercises could prevent the reduction of MIO over the short (5–10 weeks) and long‐term (3 months) [15]. We did not find any baseline variable associated with reduced risk for trismus. This finding could be due to the fact that the incidence of trismus was quite low in this cohort [16]. It seems that the risk of reduced MIO post RT has little association with psychosocial factors at baseline and depends more on the maintaining of the jaw function during treatment.

In this study, the patient‐reported outcomes for psychosocial functioning were generally between low and moderate, and general health was perceived as good to very good. In patients with TMD, it is well known that the influence of psychosocial factors such as personality, behaviour, and environment influences the onset and development of symptoms [1]. Our population had recently been diagnosed with a malignant disease, so one could expect a higher level of anxiety or depression in this population. Patients with a malignant disease often experience depression and anxiety [38]. Studies have suggested that rates of depression in patients with HNCs increase after cancer treatment. About 33% of patients experience clinically significant symptoms of depression after RT, whereas only about 15% of patients experience clinically significant symptoms of depression before RT [39]. Although the prevalence of TMD diagnosis at baseline was relatively high in this study population (39%), the diagnosis of TMD associated with psychosomatic self‐reported symptoms seems to be low. This finding may be related to the fact that pain in the masticatory system in HNC patients before treatment could be caused by the malignant disease itself. That is, the pain could be related to the tumour infiltration site, size and stage [40] rather than psychosocial factors known for development in the general population [1]. Cancer pain arises through a variety of mechanisms. In HNC, pain results from intricate interactions among the cancer itself, its microenvironment, and the systemic physiological processes involved at different stages of carcinogenesis. These dynamic interactions persist and adapt throughout the progression of the disease, treatment phases and survivorship. This evolving nature may account for the changes in clinical pain characteristics observed at different stages of the disease [41]. Other factors relevant for predicting TMD in the general population, such as age and gender, do not seem to be predictors in this population [8].

Since jaw exercise directly targets the musculoskeletal structures involved in TMD, its predictive power in pain reduction and the prevalence of myalgia likely overshadows other variables, which may have secondary or indirect effects. Other predictors, such as psychological factors or general health status, may contribute to pain modulation. However, when included in the regression model, their influence was relatively minor compared to the effect of jaw exercises. Pain management remains a critical aspect of post‐treatment care in HNC patients, particularly after RT [42]. Standard pain management strategies to alleviate mucosal pain typically involve the use of analgesics such as nonsteroidal anti‐inflammatory drugs and opioids as well as adjuvant therapies such as corticosteroids and topical agents [43]. Furthermore, physical therapies, including jaw exercises, can play a vital role in reducing pain and improving function in HNC patients undergoing RT [44]. Since effective pain management not only helps reduce discomfort but also supports rehabilitation and recovery following RT, more scientific research is necessary to improve pain management during and after radiation therapy [45].

### 5.1 Strengths and Limitations

This study benefits from the use of a range of validated instruments included in the DC/TMD for psychosocial assessments (e.g., PHQ‐9 and GAD‐7), a tactic that enhances the reliability of the data collected. Furthermore, examinations were blinded to group allocation, reducing the risk of examiner bias and a robust set of statistical analyses, including univariable and multivariable tests, ensures the comprehensive evaluation of the data.

However, several limitations must be acknowledged. First, the study was conducted at a single institution, which may limit the generalisability of the results to other settings or populations. Moreover, although the study focuses on TMD symptoms and their relationship to jaw function and psychosocial factors, other potentially important outcomes such as xerostomia (dry mouth) and overall dental health are not addressed. The reliance on self‐reported measures for psychosocial outcomes such as depression, anxiety, and stress could also introduce response biases, which may affect the accuracy of these assessments. Finally, although the study controlled for several baseline variables, other unaccounted confounders such as psychological resilience or social support could have influenced the outcomes and introduced additional variability. Additionally, the use of pain‐modulating medications was not systematically recorded in the present study. This represents a limitation, as such medications could potentially act as confounding factors or even serve as predictive markers for chronic pain outcomes.

### 5.2 Conclusion

This study concludes that jaw exercise given during RT is the main factor that reduces the risk of developing myalgia and the degree of pain. Therefore, we recommend a preventive jaw training programme be implemented for patients at risk since it may prevent the deterioration of MIO and development of TMD. As improved pain management post‐treatment is crucial for effective rehabilitation and recovery following RT, additional scientific studies are needed to address this area.

## Author Contributions

All authors contributed to the study conception and design. Preparation was performed by Ellie Saghafi and Lisa Tuomi. Data collection was performed by Ellie Saghafi and Kalid Kadhim. Analysis was performed by a statistician in collaboration with Ellie Saghafi and Lisa Tuomi. The first draft of the manuscript was written by Ellie Saghafi. All authors contributed to the writing and commented on previous versions of the manuscript and read and approved the final manuscript.

## Ethics Statement

The study was approved by the Swedish Ethical Review Authority, reference number (1151–18 and 2019‐00752).

## Consent

All study participants provided oral and written informed consent.

## Conflicts of Interest

The authors declare no conflicts of interest.

## Peer Review

The peer review history for this article is available at https://www.webofscience.com/api/gateway/wos/peer‐review/10.1111/joor.70019.

## Data Availability

The authors have nothing to report.
